# Oil, you’re kidding me!

**DOI:** 10.3205/oc000115

**Published:** 2019-07-05

**Authors:** James Austin, Channa Vasanth Nadarajah

**Affiliations:** 1 Hampshire Hospitals Foundation Trust, Basingstoke, United Kingdom

## Abstract

A 47-year-old female collapses with loss of consciousness secondary to septic shock and is found to have a left orbital fracture and a right orbital mass that was not seen on imaging two years ago. The mass is reported as a possible ocular melanoma. She is free of any eye pain, ophthalmoplegia or deterioration of vision but has pre-existing extensive diabetic proliferative retinopathy. One year ago, she underwent delamination and segmentation with silicone oil injected as a tamponade agent into the right eye. She is seen promptly in the ophthalmological clinic where fundoscopy reveals this mass to be the silicone oil and no further action is required.

## Case description

A 47-year-old female presented to the emergency department after developing loss of consciousness with amnesia following several days of right loin pain and fevers. She awoke with left sided facial pain and a dull headache. She could not recall the prior events of the past day but was aware of blood stains on her bathroom floor. She did not report any eye pain or visual loss. 

She had a previous ischaemic stroke and was being managed for hypertension and type 1 diabetes with secondary nephropathy requiring line dialysis. She has bilateral proliferative diabetic retinopathy and is registered as blind. Her left eye had undergone panretinal photocoagulation, delamination, and vitrectomy with internal air tamponade. Her right eye had undergone delamination and segmentation with silicone oil tamponade. All procedures occurred within the previous two years.

She was taking insulin and dual antiplatelet therapy. She lived alone and was independent with her activities of daily living.

On examination, there was bilateral periorbital bruising but no ophthalmoplegia. Blood pressure was 159/102 mmHg. Ophthalmology examination showed bilateral conjunctival haemorrhages in fundoscopy with no ophthalmoplegia. Gross examination of colour and visual fields was normal.

**Figure 1:**
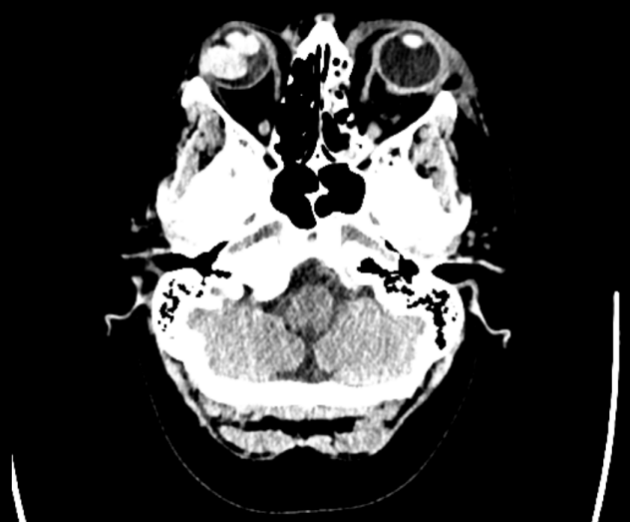
Computerised tomography of the head without contrast revealing a right hyperdense orbital mass, fracture of the left orbit with opacification of adjacent ethmoid air cells, air-fluid level in the left maxillary sinus, and subcutaneous emphysema with bruising overlying the left orbital region

A CT brain without contrast was performed revealing a right-sided hyperdense orbital mass of 2.2 x 1.9 cm (Figure 1 (Fig. 1)). A fracture of the medial wall of the left orbit with opacification of the adjacent ethmoid air cells, subcutaneous emphysema and an air-fluid level in the left maxillary sinus were also present. These findings were not present on a previous CT brain two years ago. Her blood tests showed a raised CRP and a mid-stream urine culture showed a pyuria with no growth. Given the uncertain source, a CT abdomen/pelvis was performed which revealed a small pleural effusion and anterior abdominal wall fat stranding secondary to trauma but no focal signs of infection.

The patient was reviewed in the clinic where direct fundoscopy by the ophthalmologist visualised a layer of silicone oil and this corresponded with the imaging findings.

The hyperdense mass corresponded to the silicone oil used as a tamponade agent making this an incidental finding and no further treatment was required.

Her raised CRP and back pain were suggestive of pyelonephritis and she completed a course of co-amoxiclav. She made a full recovery and was discharged home.

## Discussion

A radiopaque mass in the orbit can represent a number of possibilities including neoplasms both malignant and benign, haematoma, cysts or a subchoroidal abscess. 

It is known that silicone oil has high attenuation on CT imaging of 113–130 HU [1] and it is increasingly replacing gas mixtures as the internal tamponade agent of choice [2]. It can be placed permanently or removed at a later date. The finding of silicone oil on CT is recognised within ophthalmology but CT head scans are used across many specialities bringing other clinicians frequently in contact with these and other findings in the orbit. Previous reports record silicone oil mimicking vitreous haemorrhage [3] and it can migrate even entering the optic nerve and ventricular system [4].

## Conclusion

Medical treatments and procedures should be considered in the differential diagnosis when a new lesion is detected on imaging. Clinicians other than ophthalmologists who frequently perform CT head scans will regularly encounter findings in the orbit and should have an awareness of these differentials. For clinicians outside of ophthalmology, it may be helpful that correspondence from ophthalmologists makes a note of the appearance on silicone oil on any future cranial imaging that takes place.

## Notes

### Competing interests

The authors declare that they have no competing interests.
